# Development and function of regulatory innate lymphoid cells

**DOI:** 10.3389/fimmu.2022.1014774

**Published:** 2022-10-04

**Authors:** Christopher M. Thomas, R. Stokes Peebles

**Affiliations:** ^1^ Division of Allergy, Pulmonary, and Critical Care Medicine, Vanderbilt University Medical Center, Nashville, TN, United States; ^2^ Department of Pathology, Microbiology, and Immunology, Vanderbilt University School of Medicine, Nashville, TN, United States; ^3^ Research Service, Tennessee Valley Healthcare System, United States Department of Veterans Affairs, Nashville, TN, United States

**Keywords:** regulatory, innate, lymphoid, IL-10, cell

## Abstract

Innate lymphoid cells (ILCs) are a critical element of the innate immune system and are potent producers of pro-inflammatory cytokines. Recently, however, the production of the anti-inflammatory cytokine IL-10 has been observed in all ILC subtypes (ILC1s, ILC2s, and ILC3s) suggesting their ability to adopt a regulatory phenotype that serves to maintain lung and gut homeostasis. Other studies advocate a potential therapeutic role of these IL-10-expressing ILCs in allergic diseases such as asthma, colitis, and pancreatic islet allograft rejection. Herein, we review IL-10 producing ILCs, discussing their development, function, regulation, and immunotherapeutic potential through suppressing harmful inflammatory responses. Furthermore, we address inconsistencies in the literature regarding these regulatory IL-10 producing ILCs, as well as directions for future research.

## Introduction

Innate lymphoid cells (ILCs) are an immune cell type that have cytokine production features of T lymphocytes but lack rearranged antigen receptors. As a result, ILCs lack antigen specificity and instead respond to alarmins released predominantly, but not exclusively, by epithelial and endothelial cells in response to damage caused by infection, injury, or disease. Currently, three groups of ILCs have been discovered and defined. Group 1 innate lymphoid cells (ILC1s), the counterpart to CD4^+^ T helper (Th) type 1 cells, produce interferon gamma (IFN-γ) and express the transcription factor T-bet (1, 2). Group 2 Innate Lymphoid cells (ILC2s), analogous to CD4^+^ Th2 cells, produce interleukin (IL)-5, IL-9, and IL-13, and express the transcription factor GATA binding protein 3 (GATA-3) (3–5). Group 3 innate lymphoid cells (ILC3s), that parallel CD4^+^ Th17 cells, produce IL-17 and IL-22, and express the transcription factor retinoid-related orphan receptor gamma t (RORγt) (6–9).

**Figure 1:**
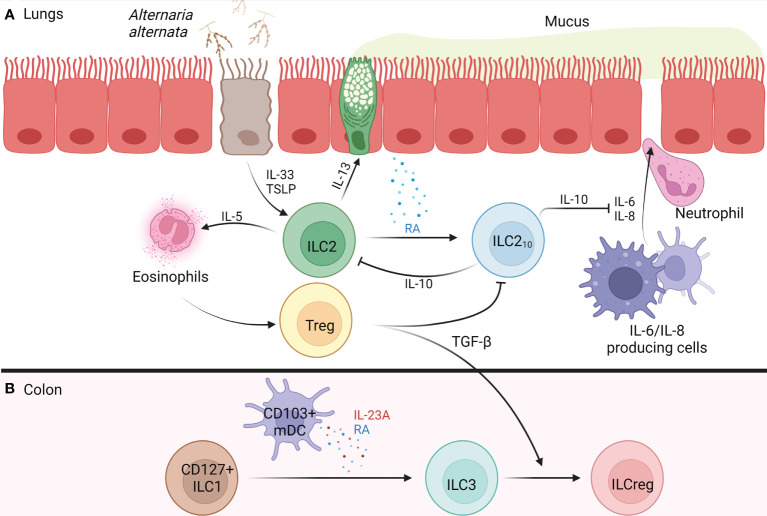
Development of IL-10^+^ ILCs in the Lung and Colon of Humans. **(A)** *Alternaria alternata* activates airway epithelium 2a) activated airway epithelium releases TSLP and IL-33 2a) IL-33 activates ILC2s, causing release of IL-5 and IL-13, while TSLP confers corticosteroid resistance 3a) IL-5 recruits and activates eosinophils 4a) IL-13 causes goblet cell hyperplasia, AHR, and release of RA from airway epithelium 5a) RA promotes the trans differentiation of ILC2s into ILC2_10_s 6a) ILC2_10_s release IL-10 which inhibits ILC2-mediated type 2 inflammation and maintains barrier integrity through the inhibition of IL-6 and IL-8 which function to increase barrier permeability, resulting in neutrophil transmigration 7a) Tregs form and regulate type 2 inflammation through release of TGF-β which blocks ILC2_10_s. **(B)** In the colon, CD103^+^ mDCs release RA and IL-23A, promoting CD127^+^ ILC1s trans differentiation into ILC3s 2b) Tregs release TGF-β promoting the trans differentiation of ILC3s into ILCregs. AHR, airway hyperreactivity; ILC, innate lymphoid cell; ILC1, type 1 innate lymphoid cell; ILC2, type 2 innate lymphoid cell; ILC3, type 3 innate lymphoid cell; ILC2_10_s, IL-10^+^ type 2 innate lymphoid cell; ILCreg, regulatory innate lymphoid cell; IL, interleukin; mDC, monocyte-derived dendritic cell; RA, retinoic acid; TGF-β, transforming growth factor beta; Treg, regulatory T cell; TSLP, thymic stromal lymphopoietin. **Figure 1** was created using BioRender.com.

In the field of allergy, ILC2s are a primary focal point due to their double-edged sword nature in both the pathogenesis, and possibly prevention, of allergic disease. In the respiratory and gastrointestinal tracts, epithelial cells can be challenged by infectious agents or allergens that contain pathogen- or damage- associated molecular patterns, resulting in epithelial cell release of alarmins: IL-25, IL-33, and thymic stromal lymphopoietin (TSLP), which activate ILC2s (10–12). ILC2s respond by migrating to the challenged site where they proliferate and release the pro-inflammatory cytokines mentioned earlier at an amount that is 10-fold greater, on a per cell basis, than that released by their CD4^+^ Th2 counterpart (13). As a result, ILC2s can participate in host protective roles, such as the eradication of helminthic parasites through IL-5-induced eosinophil recruitment and IL-13-induced goblet hyperplasia and peristalsis (14–16). However, when ILC2s are activated by alarmins in the setting of asthma, the IL-5 they produce can lead to eosinophil activation whose products damage the airway and exacerbate bronchoconstriction. IL-13 is a central mediator of asthma by promoting bronchial hyperresponsiveness and airway remodeling, as shown in **Figure 1** (17). Additionally, IL-13 disrupts the integrity of the epithelial barrier by breaking down tight junctions (18) and promoting TSLP release, leading to corticosteroid resistance in ILC2s (19).

To promote immunologic tolerance, T regulatory cells (Tregs) derived in the thymus or extrathymically from CD4^+^ naïve T cells release the anti-inflammatory cytokines transforming growth factor beta (TGF-β) and IL-10 (20, 21). Interestingly, recent studies reveal a unique ability for ILCs to adopt a regulatory phenotype, similar to Tregs, through production of IL-10. Herein we review the development, function, regulation, pathogenic and potential immunotherapeutic roles of IL-10-producing ILCs, as well as address controversies and directions for future research.

## Regulatory innate lymphoid cells (ILCregs)

Using IL-10-green fluorescent protein (GFP) reporter mice, a small subset of Lin^-^ CD45^+^ CD127^+^ IL-10^+^ ILCs were identified in the small intestinal lamina propria (sLP) at baseline. Sample analysis of human intestinal biopsies using flow cytometry also confirmed the presence of these IL-10^+^ ILCs in the sLP of humans at baseline (22). These cells were named regulatory innate lymphoid cells (ILCregs) due to their absence of ILC1 markers NK1.1, NKp46, and *Tbx21* (encodes T-bet); ILC2 markers ST2, killer cell lectin-like receptor subfamily G member 1 (KLRG1), and GATA-3; and ILC3 markers NKp46, CD4, and RORγt. Thus, these IL-10^+^ ILCs were deemed to be a new kind of ILC subset (22).

Interestingly, while ILCregs exhibited similarities to Tregs, such as their ability to produce IL-10 and TGF-β, they lacked expression of the Treg transcription factor Foxp3 (23). Unlike ILC1s, ILC2s, and ILC3s, ILCregs originate from the common helper-like innate lymphoid precursor (CHILP)-α4β7^+^Id2^high^ and express *Id3* which is required for their development/maintenance (1, 22). Due to the constitutive presence of ILCregs in the intestines and their expansion seen during dextran sodium sulfate (DSS)-induced colitis in Rag^-/-^ mice (22), ILCregs have been conjectured to maintain gut tolerance through production of IL-10. When activated ILC1s and ILC3s were adoptively transferred into DSS-induced colitis Rag1^-/-^ *Il2rg^-/-^* (ILCreg deficient) mice, severe colitis resulted, an effect that was attenuated upon ILCreg reconstitution (22). However, severe colitis resulted upon transferring IL-10Rα^-/-^ ILC1s and ILC3s into Rag1^-/-^ *Il2rg^-/-^* mice reconstituted with WT ILCregs, revealing that ILCregs protect against colitis through IL-10 (22). Notably, Tregs isolated from *Foxp3*-DTR (human diphtheria toxin receptor)-GFP mice adoptively transferred into ILC1/ILC3 reconstituted Rag1^-/-^ *Il2rg^-/-^* mice had no effect on intestinal inflammation even after the depletion of Foxp3^+^ Tregs using diphtheria toxin (DT) treatment (22). However, when ILCreg^DTR^ cells were depleted in the intestines of mice following DT treatment, severe inflammation ensued (22). Importantly, these studies distinguish ILCregs as having a unique protective function in the intestines of mice.

In addition to the sLP, ILCregs have been discovered residing in the kidney’s interstitium of both humans and mice at baseline. These ILCregs produce large amounts of IL-10 and TGF-β that protect against renal ischemia/reperfusion injury (IRI), an effect that was abolished by neutralizing IL-10 and TGF-β antibodies (24). Interestingly, administration of an IL-2/anti-IL-2 monoclonal antibody complex (IL-2c) expanded ILCregs in the kidney of IRI Rag^-/-^ mice, reducing tubular epithelial cell apoptosis and improving renal function (24). Importantly, depletion of these renal ILCregs using PC61 (an anti-CD25 antibody) showed greater kidney injury in IRI Rag^-/-^ mice, revealing their critical role in renal protection (24). Adoptive transfer of ILCregs expanded *ex vivo* with IL-2c into IRI C57BL/6 mice further confirmed their protective role by restoring kidney function through the suppression of ILC1 and neutrophil infiltration and enhancing M2 macrophage generation (24). Notably, ILCregs in the kidneys reduced the frequency of ILC1s but not ILC2s or ILC3s, suggesting a pathogenic role of ILC1s in renal IRI (24) as well as differences in ILCreg function dependent on anatomical location (see **Table 1**).

**Table 1:** Differences between mouse and human ILCregs and ILC2_10_s.

Cell	Location (Ref)	Species	Phenotype	% Of Total IL-10^+^ ILCs at Baseline	Express *Id3*	Express GATA-3/KLRG1/ST2?	TGF-β	Function
ILCregs	Kidneys (24)	Human	Lin^-^ CD127^+^ CD161^+^ IL-10^+^	~4.4%	Yes	No	Stimulatory	Suppresses ILC1s
		Mouse	Lin^-^ CD127^+^ IL-10^+^	~2.7%				
	Intestines (22)	Human	Lin^-^ CD45^+^ CD127^+^ IL-10^+^	~15%				Suppresses ILC1s and ILC3s
		Mouse		~13%				
ILC2_10_s	Lungs (25,26)	Human	Lin^-^ CD45^+^ CD127^+^ CD161^+^ IL-10^+^	0%	No	Yes	Inhibitory	Suppresses ILC2s
		Mouse	Lin^-^ CD45^+^ Thy-1.2^+^ IL-10^+^	~0.4%				

GATA-3, GATA binding protein 3; Id3, inhibitor of DNA binding 3; ILCs, innate lymphoid cells; ILC1s, type 1 innate lymphoid cells; ILC2s, type 2 innate lymphoid cells; ILC3s, type 3 innate lymphoid cells; ILC210s, IL-10+ type 2 innate lymphoid cells; ILCregs, regulatory innate lymphoid cells; KLRG1, killer cell lectin-like receptor G1; Ref, reference; sLP, small intestine lamina propria; ST2, soluble interleukin 1 receptor-like 1; TGF-b, transforming growth factor beta.

## Regulatory phenotype of ILC2s

There is also evidence that ILC2s have the capacity to produce IL-10 and may have immunoinhibitory potential. For instance, the hypoxic microenvironment of pancreatic ductal adenocarcinoma tumors (PDAC) can promote ILC2s to become regulatory IL-10^+^ ILC2s through the upregulation of hypoxia-inducible factor 1-alpha (HIF-1α) which binds to the *Il10* promoter (27). Importantly, reoxygenation or neoadjuvant chemotherapy caused IL-10^+^ ILC2s to convert back into ILC2s, suggesting a regulatory plasticity. Unlike the previously described ILCregs, IL-10^+^ ILC2s maintained their ILC2 phenotype through the expression of Il1rl1 (ST2), KLRG1 (26) and *Gata3* (28); thus, they have been termed ILC2_10_s (see **Table 1**).

ILC2s treated with the common Treg polarization factors TGF-β, vitamin D, or retinoic acid (RA), became ILC2_10_s only in the presence of RA (25). Notably, administration of a pan- retinoic acid receptor (RAR) inhibitor blocked ILC2_10_s generation in a dose-dependent manner, revealing that RA acts through RAR to induce the ILC2_10_ phenotype (25). In an *in vitro* study using air liquid interface (ALI) cultures of primary bronchial epithelial cells treated with IL-5, IL-13, and IL-33, from patients with chronic rhinosinusitis with nasal polyps (CRSwNP), only IL-13 promoted RA generation (25). This result suggests that IL-13 derived from ILC2s upregulates ILC2_10_s by promoting RA generation from epithelial cells, which in turn downregulates the ILC2-induced type 2 inflammatory response through IL-10 release (see **Figure 1**). This implies that ILC2s have a mechanism to autoregulate the inflammation that they induce.

In a model of allergic lung inflammation in mice induced by either four daily intranasal administrations of IL-33 or chronic papain exposure, a population of IL-10 producing Lin^-^ Thy1.1^+^ ILC2s emerged (26). Interestingly, the same population of IL-10^+^ ILC2s was induced by treating ILC2s *in vivo* with IL-2c (26). However, ILC2 production of IL-10 is not restricted to the lungs. When treating small intestinal ILC2s from naïve mice with IL-2, IL-4, IL-10, IL-27, and neuromedin U (NMU) together, these ILC2s began producing IL-10 (29). Interestingly, IL-2 and IL-4 enhanced IL-10 production by ILC2 when these cytokines were administered individually in culture (29). As a result, these experiments collectively suggest that ILC2 trans-differentiation into ILC2_10_s is a self-amplifying process instructed by their cytokine milieu and environment.

## Regulation of IL-10 producing ILCregs and ILC2s

Immune suppression is not always beneficial, as in the case of PDAC tumors where IL-10^+^ ILCs promote tumor growth (27). As a result, regulation of IL-10 by ILCs is crucial. A study conducted on ILC2 from WT and C3a receptor knockout (C3ar^-/-^) mice reported that genetic deletion of the C3a receptor resulted in significantly less IL-13, IL-5, and granulocyte-macrophage colony-stimulating factor (GM-CSF) production, while C3a signaling inhibited IL-33-induced IL-10 production from ILC2_10_s (30). Thus, the anaphylatoxin C3a combined with IL-33 stimulation enhanced the pro-inflammatory ILC2 phenotype through inhibiting *Il10* transcription and promoted ILC2 antigen-presentation to CD4^+^ T cells, resulting in Th2 differentiation (30). Additionally, tumor necrosis factor-like cytokine 1A (TL1A) strongly abrogated IL-10 production in ILC2_10_s while increasing IL-5 and IL-13 production (29). Collectively, these results reveal that the regulatory phenotype adopted by ILC2s is reversible and influenced by environmental conditions.

Cytokines can also downregulate IL-10-expressing ILCs. In human ILC2_10_s from patients with systemic sclerosis (SSc), treatment with TGF-β dramatically decreased the production of IL-10 and reduced KLRG1 expression, an ILC2 surface marker found to be required for IL-10 production (29, 31, 32). However, unlike ILC2_10_s, ILCregs rely on TGF-β signaling for their survival and expansion (see **Table 1**), as seen through the effects of deleting TGF-β receptors on ILCs using Tgfbr2^flox/flox^;CreERT2 mice (22, 33). This finding reveals differences between ILCregs and ILC2_10_s, potentially revealing the presence of two regulatory ILC subtypes.

## Regulatory phenotype of ILC3s and ex-ILC1s

Several pieces of evidence suggest that ILC3s are plastic and can become ILCregs. A study investigating colorectal cancer (CRC) tumor infiltrating ILCs from azoxymethane/dextran sodium sulfate (AOM/DSS)-induced colitis models revealed that ILC3 numbers decreased, while ILCreg numbers increased, during CRC tumor progression (34). At the late-stage of CRC tumors, fate mapping using Rosa26-STOP-tdTomato;Rorc-Cre;IL-10-GFP lineage tracing mice followed by AOM/DSS treatment revealed former ILC3s (exILC3s) producing IL-10 and expressing *Id3* (34). Using TGF-β receptor knockout mice treated with AOM/DSS, ILCreg numbers decreased while ILC3 numbers increased, causing tumor growth suppression (34). Furthermore, ILC3 treatment with a TGF-β inhibitor prevented the conversion of ILC3s to ILCregs, a result that was consistent in both the AOM/DSS-induced CRC mice and patient derived xenograft (PDX) tumors (34). Collectively, TGF-β drives the trans-differentiation of ILC3s towards ILCregs in both humans and mice. This important finding reveals that IL-10 production from ILCs is not limited to KLRG1^+^ ILC2s, as previously thought (29, 32), and brings to question whether ILC1s can adopt a regulatory phenotype.

CD127^+^ ILC1s that lost their ability to proliferate contained the capacity to reversibly differentiate into ILC3s (exILC1s) in the presence of IL-2, IL-23, and IL-1β when administered together (35). Further analysis revealed that exILC1s lost their T-bet expression and IFN-γ production, but began expressing RORγt and producing IL-22, committing to an ILC3 phenotype (35). Notably, in the presence of IL-2 and IL-12, ILC3s and exILC1s lost their RORγt and IL-22 expression while upregulating T-bet expression and IFN-γ production, committing to an ILC1 phenotype (35). In addition to the mentioned cytokines, RA signals through its receptors (RARA, RARG, and RXRG) present on CD127^+^ ILC1s to accelerate the differentiation of ILC1s into ILC3s (35, 36). Human monocyte derived dendritic cells (mDCs) treated with RA upregulated CD103 expression and began producing RA and IL-23A under basal conditions and lipopolysaccharide (LPS) stimulation, revealing a role CD103^+^ mDCs play in CD127^+^ ILC1s differentiation toward ILC3s (35). As a result, it is possible ILC1s can become ILCregs through their commitment to an ILC3 phenotype in the presence of CD103^+^ mDCs (**Figure 1**). However, *ex vivo* stimulation with IL-12/IL-15 markedly increased IL-10 production in human ILC1s revealing their direct ability to adopt a regulatory phenotype (33). These findings reveal a regulatory plasticity within all ILC subtypes, and potential crosstalk between DCs and ILCs which should be further investigated in future research.

## Immunotherapeutic potential of IL-10 producing ILCs through stimulation or inhibition

Through *in vivo* generation and stimulation in the lungs, ILC2_10_s show promise as potential therapeutics for allergic airway inflammation. Using CRSwNP patient nasal epithelial cells, ALI cultures co-cultured with ILC2_10_s and challenged with grass-pollen allergen revealed that the addition of the ILC2_10_s prevented allergen-induced epithelial barrier disintegration, an effect that was diminished upon the addition of anti-IL-10 neutralizing antibodies (32). Elevation of IL-10R surface expression on epithelial cells occurred upon allergen exposure, enhancing the ILC2_10_-induced epithelial barrier restoration (32). As a result of this restoration, grass-pollen sublingual allergen immunotherapy (GP-SLIT) was investigated in allergic individuals. In groups treated with GP-SLIT, frequencies of ILC2_10_s increased compared to the placebo-treated group, negatively correlating with clinical symptoms (32). This result shows promise in using GP-SLIT to induce ILC2_10_s in atopic individuals, which function to restore barrier integrity and attenuate type 2 inflammation through IL-10 production. Furthermore, an *in vitro* study on nasal epithelium from allergic individuals co-cultured with ILC2_10_s revealed that IL-10 served to maintain epithelial and endothelial barrier integrity by blocking IL-6 and IL-8, both of which promote neutrophil translocation by increasing barrier permeability as shown in **Figure 1** (37, 38). In mice, IL-10 attenuated Th2-mediated allergic airway inflammation by downregulating Th2 survival through restoring granzyme B expression in CD4^+^ IL-10^-/-^ cells (39).

To further investigate the immunosuppressive role of ILC2_10_s in allergic diseases such as asthma, ILC2_10_s and ILC2s in a 1:1 mix were adoptively transferred into Rag^-/-^ γc^-/-^ (T-cell, B-cell, and NK cell deficient) mice intranasally challenged with IL-33. In doing so, ILC2-dependent allergic airway hyperreactivity (AHR) was downregulated, a result that was abrogated upon the intraperitoneal administration of anti-IL-10R (40). ILC2_10_s-induced AHR attenuation was further confirmed in mice challenged with *Alternaria alternata* that were adoptively transferred the same 1:1 ILC2_10_s/ILC2s mix. The role of IL-10 was confirmed when administration of anti-IL-10R antibodies abrogated this effect (40). Collectively, *in vivo* generation of ILC2_10_s in the lungs attenuates type 2 allergic responses through IL-10 production.

Another potential therapeutic role of ILC2_10_s is the promotion of islet allograft survival in mice as measured through improved glucose tolerance (41). ILC2_10_s were delivered to recipient mice either intravenously or through co-transplantation with the graft. Interestingly, allograft survival was increased in only the co-transplantation group, revealing a need for ILC2_10_s to be within the graft to achieve maximal graft protection (41). Further investigation is needed to determine how these findings translate into clinical practice.

## ILCregs and ILC2_10_s – The same cell or are they different?

In this review we discussed ILCregs as those cells that express *Id3*, are stimulated by TGF-β, and arise from the α4β7^+^Id2^high^ CHILPs or from ILC3s in the presence of TGF-β. Separately, we defined IL-10^+^ ILC2s as ILC2_10_s as a consequence of their sustained expression of GATA-3 and suppression by TGF-β (see **Table 1**). However, whether these cells are the same or different remains to be fully defined. Notably, ILCregs arose in the gut and kidneys at steady state and during inflammation (22), while ILC2_10_s arose in both the gut and lungs in the presence of inflammation only (25, 27). As such, further studies should be directed towards the molecular comparison of ILCregs and ILC2_10_s to determine if their GATA-3 expression and response to TGF-ß is cell type specific or influenced by their environment/location.

As previously discussed, ILCregs devoid of all ILC markers were expressed in the sLP of mice (22). However, upon repeat of this experiment by a different group, no such cell population was found (29). Interestingly, this group discovered that only Lin^-^ CD127^+^ Thy1^-^ ILC2s expressed IL-10 in the small intestine (29). This finding revealed inconsistencies surrounding the presence and identification of ILCregs in the sLP. One reason for the inconsistent result was suggested to be caused by genetics and/or environmental factors. However, even controlling for these factors by purchasing C57BL/6 mice from three different vendors, no ILCregs were identified (29). As a result, the existence of ILCregs in mice are non-generalizable. Further studies need to investigate the contributions of other environmental influences such as inflammation or autoimmunity on the presence of ILCregs, in both the intestines of mice and humans.

Through studying the suppressive function of ILCregs in a mouse model of colitis, IL-10 inhibited the activation of both ILC1s and ILC3s, as previously discussed. However, in an *in vitro* study investigating the suppressive role of TGF-β and IL-10 in human ILC subsets, IL-10 inhibited cytokine production from pre-stimulated ILC2s while having no effect on pre-stimulated ILC1s (33). As a result, further studies are needed to determine the differential role of ILCregs in repressing the function of ILC1s, ILC2s, and ILC3s between mice and humans, and to determine whether this difference is influenced by the inflammatory environment.

Other roles of ILC2_10_s remains to be investigated, such as its ability to suppress lung eosinophilia. Through treating Rag^-/-^ mice with IL-33 and IL-2c, a significant reduction in IL-33-induced lung eosinophilia occurred with extensive generation of ILC2_10_s (26). However, no inhibitory studies using anti-IL-10 antibodies or IL-10^-/-^ ILC2s were performed to prove the role of ILC2_10_s in attenuating eosinophil migration to the lungs. As a result, *in vivo* delivery of IL-2c should be further investigated in its efficacy as an immune-targeted therapy that could reduce eosinophilia in atopic patients as well as protect against renal IRI, colitis, allergic airway inflammation, and allograft rejection due to its ability to generate ILC2_10_s both *in vivo* and *in vitro*.

Interestingly, a cross-sectional study comparing grass-pollen allergic (GPA) and house dust mite-allergic (HDMA) individuals to a non-atopic healthy control (NAC) revealed that ILC2s from atopic individuals fail to adopt an IL-10-producing regulatory phenotype (32). This finding reveals a possible limitation in treating allergic disease through ILC2_10_ generation. As a result, the regulation of the IL-10 promoter in ILC2s from GPA and HDMA patients should be investigated as it could further explain the lack of immune regulation seen in atopic patients.

## Conclusion

There is increasing evidence that the IL-10 produced by ILCs suppresses immune responses and could be helpful, such as in allergic disease, or harmful, such as in the setting of cancer, to patients. However, due to the limitations regarding the specific deletion of IL-10^+^ ILCs *in vivo*, these cells remain an enigma as their exact role in human or mouse disease remains unknown. For instance, there are no specific surface markers for ILCregs for which antibody depletion could target to determine their role in regulating inflammatory processes. This is an emerging field that is certainly ripe for further investigation to understand the full nature and importance of these suppressive ILCs in human health.

## Author contributions

CT wrote all drafts of the manuscript and RP edited the manuscript drafts. All authors contributed to the article and approved the submitted version.

## Funding

This study was supported by NIH/NIAID 5RO1 AI124456, 5RO1 AI145265, 5R21 AI145397, 2U19 AI095227, and U.S. Department of Veterans Affairs 5I01BX004299

## Conflict of interest

The authors declare that the research was conducted in the absence of any commercial or financial relationships that could be construed as a potential conflict of interest.

## Publisher’s note

All claims expressed in this article are solely those of the authors and do not necessarily represent those of their affiliated organizations, or those of the publisher, the editors and the reviewers. Any product that may be evaluated in this article, or claim that may be made by its manufacturer, is not guaranteed or endorsed by the publisher.
